# Fructo-Oligosaccharides Modify Human DC Maturation and Peanut-Induced Autologous T-Cell Response of Allergic Patients *In Vitro*

**DOI:** 10.3389/fimmu.2020.600125

**Published:** 2021-02-15

**Authors:** Simone M. Hayen, André C. Knulst, Johan Garssen, Henny G. Otten, Linette E. M. Willemsen

**Affiliations:** ^1^ Department of Dermatology/Allergology, University Medical Center Utrecht, Utrecht University, Utrecht, Netherlands; ^2^ Laboratory of Translational Immunology, University Medical Center Utrecht, Utrecht, Netherlands; ^3^ Division of Pharmacology, Utrecht Institute for Pharmaceutical Sciences, Faculty of Science, Utrecht University, Utrecht, Netherlands; ^4^ Department of Immunology, Nutricia Research B.V., Utrecht, Netherlands

**Keywords:** dendritic cells, non-digestible oligosaccharides, T cells, peanut allergy, immunomodulation

## Abstract

**Background:**

Dendritic cells (DCs) play an important role in antigen presentation, and are an interesting target for immune-modulation in allergies. Short- and long-chain fructo-oligosaccharides (scFOS/lcFOS, FF) have immunomodulatory capacities, and may influence the outcome of DC antigen presentation.

**Objective:**

This study investigated the effect of FF during DC maturation and allergen presentation using cells of peanut-allergic patients in an autologous DC-T cell assay.

**Methods:**

CD14^+^ and CD4^+^ T cells were isolated from peanut-allergic patients. CD14^+^ monocytes were differentiated into immature DCs (imDCs), and matured (matDCs) in the presence or absence of crude peanut-extract (CPE) and/or FF, and co-cultured in an autologous DC-T cell assay. T cell polarization, proliferation and cytokine production were measured.

**Results:**

Expression of maturation surface molecule markers on matDCs was not affected by CPE and/or FF. By contrast, the IL-10 secretion by matDCs increased compared to imDCs, upon exposure to CPE and FF compared to CPE alone. Also the IP-10 secretion increased in CPE/FF-matDCs compared to imDC. CPE-matDCs enhanced IL-13 release in the DC-T-cell assay and Treg polarization in presence or absence of FF. CPE/FF-DCs tended to increase the Treg/Th1 and Treg/Th2 ratios compared to matDCs. The proliferation of both Treg and Th2 cells tended to increase when T cells were co-cultured with CPE-matDCs compared to matDCs, which became significant when CPE-matDCs were also exposed to FF and a same tendency was shown for Th1 proliferation.

**Conclusion:**

Only in the presence of FF, CPE-matDCs produced increased regulatory and Th1-related mediators. CPE-matDCs modified T cell polarization and proliferation, and additional exposure to FF tended to enhance Treg/Th2 and Treg/Th1 ratios instructed by CPE/FF-matDCs. However this effect was not strong enough to suppress CPE-matDCs induced IL-13 release by Th-cells. This indicates the ability of FF to modify DC maturation in the presence of an allergen supporting a more Treg/Th1 prone direction of the successive allergen specific Th2 cell response.

## Introduction

Food allergies are the result of a loss of tolerance toward harmless antigens, since these antigens are recognized by the immune system as harmful. The incidence of food allergies worldwide is still increasing, and antigen-specific immunotherapy strategies are still developing. Until now, little data is available about the induction of long-lasting unresponsiveness toward specific food allergens, although there are studies showing the induction of unresponsiveness in part of the patient population (1). More interest is gained toward the use of adjuvants, such as pro- and prebiotics during these forms of immunotherapy to improve existing protocols (2, 3). These adjuvants can amongst others influence the growth of beneficial bacteria in the gut, and can induce the release of beneficial mediators, such as mucosal tissue derived galectin-9 or microbial derived short-chain fatty acids (SCFAs). An example of such a prebiotic mixture is a mixture composed of short-and long-chain fructo-oligosaccharides (scFOS/lcFOS). Previous studies have indicated that a dietary intervention with fructo-oligosaccharides in humans indeed can increase the numbers of beneficial bifidobacteria (4, 5).

These prebiotics can exert their functions as a result of fermentation by gut bacteria and they also become available systemically (6–9). Traces of fructo-oligosaccharides and human milk oligosaccharides (HMOs) were retrieved in plasma and urine of newborns, indicating that several hundred milligrams can circulate in the blood daily (8–10). Previous research indicated that indeed a prebiotic mixture of short chain galacto- and long chain fructo-oligosaccharides (scGOS/lcFOS) was able to affect dendritic cells (DCs) of healthy donors directly (11). DCs play an important role in the development of food allergies. They are one of the most important antigen-presenting cells (APCs) which are involved in priming the innate and adaptive immune responses. DCs can take up antigens, process them into peptides and present them *via* the major histocompatibility complex (MHC) to T cells (12). This antigen presentation to T cells is an important aspect in the development of food allergies. Under certain conditions, these T cells can develop into Th2 cells, which produce cytokines, such as IL4, IL-5, and IL-13, and in turn can induce class-switching of B cells (12). These B cells start to produce antigen-specific IgE, which can opsonize the high-affinity IgE receptor FcϵRI on basophils and mast cells (13). Mast cell and basophil degranulation may occur on contact with the allergen following opsonization with IgE produced from allergen specific memory B-cells which develop as a consequence of previous allergen exposure and the instruction by allergen specific Th2 cells. However, clinical manifestations will be mild. These manifestations increase in intensity during a following contact, where specific IgE will be released by long-lived plasma cells after recognition of the allergen by these memory B-cells. Mediators, such as histamines are released, which will eventually lead to clinical symptoms, such as, swelling of the oral mucosa, urticaria, wheezing, and even fatal anaphylaxis.

To interfere with allergies, DCs would be an interesting target and oligosaccharides may alter their function. When antigen presentation to T cells is influenced, this might affect the development of allergies, by helping to prevent sensitization or restore tolerance by the induction of for instance Tregs. In addition to Tregs, Th1 cells can counterbalance over-reactive Th2 cells. The previously used scGOS/lcFOS mixture however may pose risks in patients with severe cow’s milk allergy, since scGOS is produced from cow’s milk derived lactose (14). Therefore, the prebiotic mixture scFOS/lcFOS might be an interesting alternative. The goal of this study was therefore to determine whether scFOS/lcFOS can affect antigen presentation of DCs to T cells. This was investigated with use of a peanut-specific autologous DC-T cell assay, using cells of peanut-allergic patients.

## Materials and Methods

### Study Population

From the clinic of Dermatology/Allergology at the University Medical Center Utrecht, fifteen peanut-allergic patients were recruited. Patients were considered peanut-allergic based on their history, a positive skin prick test (SPT) and double-blind placebo-controlled food challenge (DBPCFC). Patients between 18 and 65 years of age with a type I allergic reaction to peanut and a positive DBPCFC were included in the study, whereas pregnant patients or patients using systemic immunosuppressants, such as prednisone were excluded. The fifteen subjects (6 male, 9 female) that eventually were recruited were between 18 and 50 years of age, with a median of 32 years. All of the patients had a positive Skin Prick Test (diameter of at least 3 mm). The detailed demographic data, SPT, Müller score and the eliciting dose (ED) as established by DBPCFC are described previously (2). Before patients were enrolled in the study, they gave written informed consent. The study was reviewed and approved by the Ethics Committee of the University Medical Center Utrecht (NL51606.041.15).

### PBMC Isolation

100 ml blood of peanut-allergic patients was withdrawn in heparin tubes. Blood was diluted 1:1 with 1× PBS (Sigma-Aldrich, the Netherlands), followed by isolation of PBMCs using a Ficoll-Paque PLUS (GE Healthcare Life Sciences, Sweden) density gradient centrifugation (2,400 rpm, 20 min).

### Isolation of Monocytes and CD4+ T Cells

After PBMC isolation, cells were resuspended in MACS buffer (1× PBS, 2% FCS (Biowest, France) 0.1 mM EDTA (Thermofisher Scientific)). First, monocytes were isolated from this PBMC fraction by positive selection using MACS beads and a magnetic cell separator (CD14 Microbeads, Miltenyi Biotec, Germany). The flowthrough, containing CD14^−^ cells, was used to isolate CD4^+^ cells using negative selection (Miltenyi Biotec). CD4^+^ T cells were frozen using freezing medium (90% FCS and 10% DMSO (Sigma-Aldrich, the Netherlands)), and kept at −80°C until further use.

### Culture of Monocyte-Derived Dendritic Cells (moDCs)

**Figure 1:**
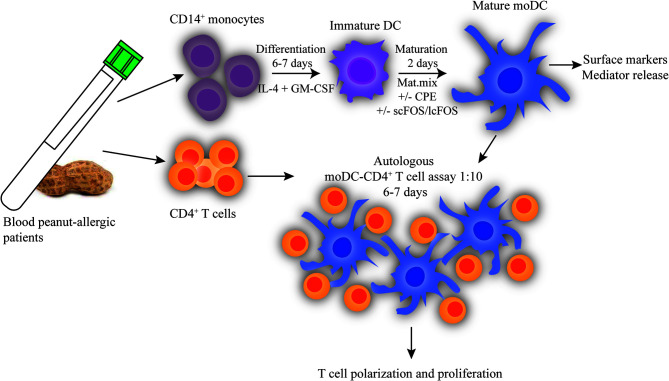
Experimental setup autologous DC-T cell assay. CD14^+^ monocytes and CD4^+^ T cells were isolated from blood of peanut-allergic patients by MACS. CD4^+^ T cells were frozen until use. CD14^+^ monocytes were differentiated with IL-4 and GM-CSF. After 6 to 7 days, immature DCs were matured for 2 days with a maturation mix (IL-6, TNF-α, IL-1β, and PGE2) either or not combined with 10 μg/ml peanut extract (CPE) and/or scFOS/lcFOS (FF). Mature moDCs were harvested and used in an autologous DC-CD4^+^T cell assay in a ratio of 1:10. CD4^+^ T cells were stained with CellTrace Violet and DCs and T cells were co-cultured for 6 to 7 days, after which T cell polarization and proliferation were determined.

CD14^+^ monocytes isolated from the PBMC fraction were brought to a concentration of 1 × 10^6^ cells/ml, and were differentiated to immature DCs (imDCs) with 20 ng/ml IL-4 and 20 ng/ml GM-CSF (both Miltenyi Biotec) in XVIVO 15 medium (Lonza, Switzerland) in a 24-wells plate (Corning Costar, Sigma Aldrich) for 6 to 7 days. Medium was refreshed at day 3 or 4. After differentiation, imDCs were matured for 2 days with a Th2-skewing maturation mix (10 ng/ml IL-1β, 10 ng/ml TNF-α, 10 ng/ml IL-6, and 1 μg/ml PGE2 (Pfizer, USA)), in presence of crude peanut-extract (CPE, 10 μg/ml) and/or scFOS/lcFOS (0.05% w/v (0.5 g/L)) (scFOS: Raftilose P95, Orafti, lcFOS: Raftiline HP, Orafti) (**Figure 1**). The CPE extract was prepared as previously described (15). In five patients (limited due to amount of patient material), the time-point of addition of scFOS/lcFOS was investigated. scFOS/lcFOS was added during the differentiation and maturation in the presence of CPE (d), or only during maturation in the presence of CPE. No differences were observed between Treg, Th2 and Th1 polarization (**Figure S1A**) and proliferation (**Figure S1B**). DCs were analyzed for expression of surface markers with use of flow cytometry. DCs were first identified as the CD14^−^ CD11c^+^ HLA-DR^+^ population. Maturation of DCs was assessed by markers CD80 (BD), CD83 (Biolegend), and CD86 (BD). Dendritic cell-specific ICAM-3–grabbing non integrin (DC-SIGN, BD) which can recognize mannose type carbohydrates was measured as possible receptor for the oligosaccharides. In addition, tolerogenic markers such as Immunoglobulin-like transcript-3 and -4 (ILT3 and ILT4, both Biolegend), PD-L1 (eBioscience), and DC2 marker OX40L were measured. After 2 days of maturation, supernatant was harvested and analyzed on the production of IL-10, IL-12, IFNα, IFNβ, CCL17, and IP-10 by means of a luminex assay. CCL17 was below the detection limit of the luminex assay. IL-12 production by DCs can promote the development of Th1 cells, while the production of type-I IFNs by DCs can increase upon Dectin-1–mediated signaling by, for example, β-glucans (16). IL-10 is a cytokine involved in tolerance and IP-10 is a Th1-related chemokine.

### Autologous DC-T Cell Assay

DCs were harvested from the wells, and washed two times with PBS. Simultaneously, CD4^+^ T cells from the same patient were thawed and washed two times with PBS. CD4^+^ T cells were stained with CellTrace Violet according to the manufacturer’s protocol (Invitrogen, USA). DCs and T cells were combined in a round-bottom 96-wells plate (Corning Costar, Sigma Aldrich) in a ratio of 1:10 in triplo. As positive control, T cells were stimulated with 1:10 CD3/28 beads (Thermo Fisher Scientific) (**Figure 1**). After 6 days, supernatant was pooled for analysis with ELISA, and T cell polarization (Th1 (CD4^+^CXCR3^+^), Th2 (CD4^+^CRTH2^+^), and Treg (CD4^+^CD25^+^CD127^−^FoxP3^+^)) and proliferation were measured with flow cytometry. Flow cytometry data were analyzed with FACS DIVA software (BD).

### ELISA

In the supernatant of the DC-T cell co-culture, IFN-γ, TNF-α, IL-10, and IL-13 were measured by ELISA, according to the manufacturer’s protocol (Ready-Set-Go, eBioscience). IFN-γ, TNF-α, and IL-10 levels were below the detection limit of the ELISA. Data analysis was performed by 4-parametric curve fitting using Microplate Manager Software.

### Statistics

Normally distributed data were analyzed by one-way ANOVA for repeated measures, with Bonferroni *post hoc* test. If the data was not distributed normally, the data was first transformed (LOG). The latter was only applied for the IL-10 and IP-10 data shown in **Figure 4**. Data were analyzed with Graphpad Prism 7.0.

## Results

### CPE Exposed matDCs Induce IL-13 Secretion by Autologous T Cells

**Figure 2:**
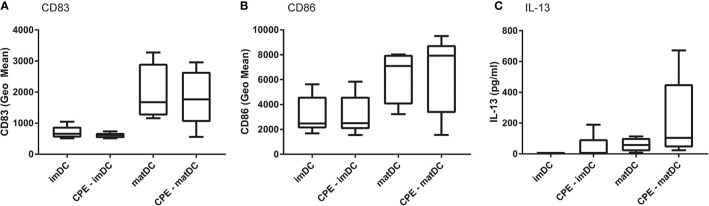
Pilot experiment to determine maturation status of DCs. In 5 patients, DCs were differentiated with IL-4 and GM-CSF, followed by incubation for 2 days with either medium (control), 10 μg/ml peanut extract (CPE), or were matured with the maturation mix, or maturation mix in combination with CPE. Expression of CD83 **(A)**, CD86 **(B)**, and IL-13 production by CD4^+^ T cells in the following autologous DC-T cell assay **(C)** wasqa determined. Data are presented as box plot summary.

To first determine the ability of DCs to present allergens to T cells, differentiated DCs of five patients were either treated with medium (control), CPE (10 μg/ml), or matured with the cytokine maturation mix (IL-6, TNF-α, IL-1β, and PGE2) either or not combined with CPE. CD83 (**Figure 2A**) and CD86 (**Figure 2B**) maturation marker expression was determined. In contrast to immature moDCs, DCs matured with the maturation mix (matDC) showed increased expression of maturation markers CD83 and CD86 in presence or absence of CPE. In addition, IL-13 production by the autologous DC-T cell co-culture was measured after 6 days of co-culture between matDCs and autologous CD4^+^ T cells. IL-13 production was absent in imDCs, and low in DCs that were solely exposed to CPE or the cytokine maturation mix. IL-13 production was highest when DCs were matured with the cytokine mix and were simultaneously loaded with CPE, indicating that maturation of the DCs is necessary to induce an autologous allergen-specific T cell response. The results presented in **Figure 2** were not significant due to the low number (five patients) and large patient variation. However based on these pilot experiment the choices for required exposures were made for the further autologous DC-T cell cultures that were performed with DCs matured with the cytokine maturation mix using blood of a second cohort of n=12 independent peanut allergic patients.

### Exposure of DCs to scFOS/lcFOS or CPE During Maturation Does Not Alter the Surface Expression of Maturation Markers

**Figure 3:**
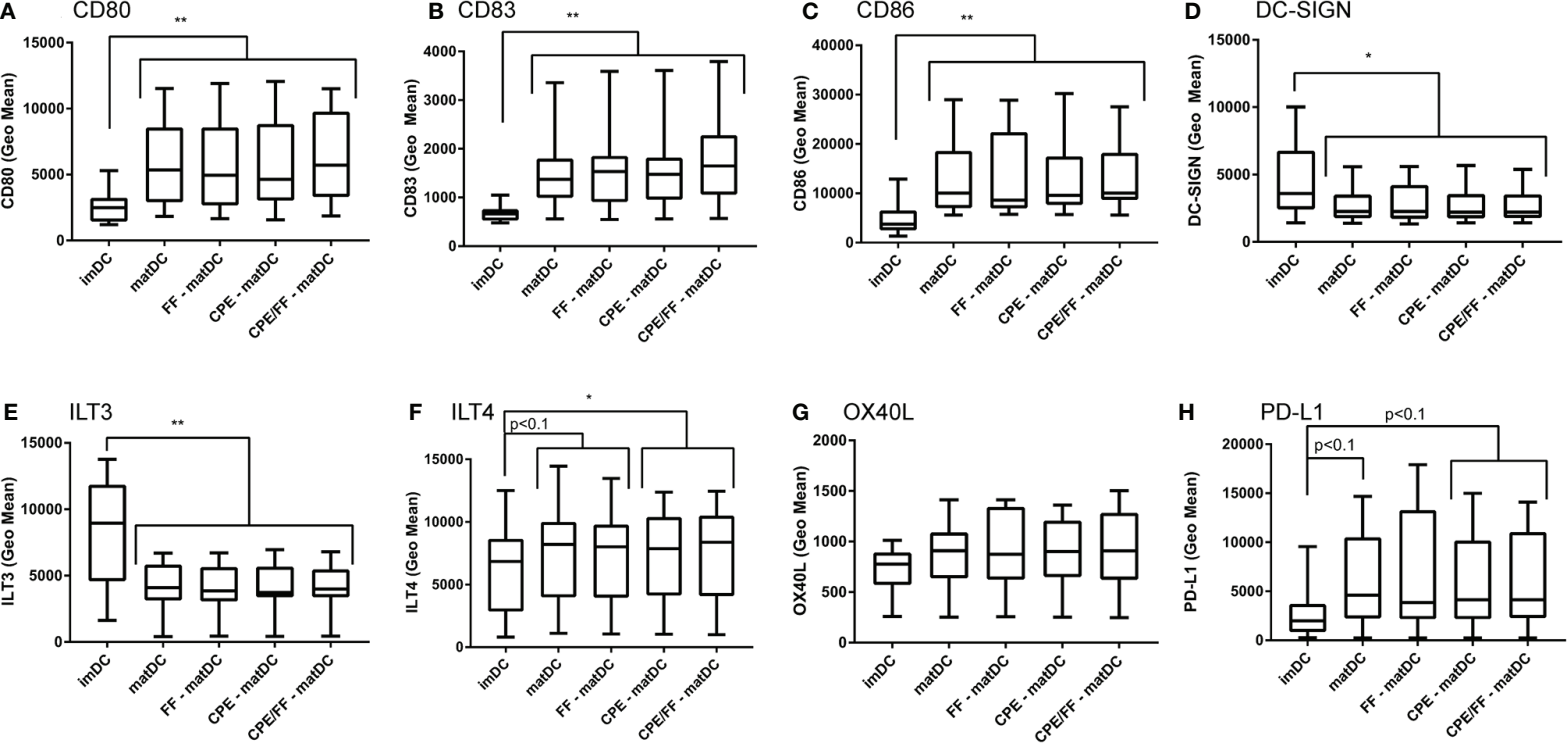
Maturation status of DCs in the presence of CPE and scFOS/lcFOS. Compared to a control sample treated with medium, DCs matured with the maturation mix containing IL-6, TNF-α, IL-1β, and PGE2 showed an increase in expression of CD80 **(A)**, CD83 **(B)**, CD86 **(C)**, while DC-SIGN **(D)** and ILT3 **(E)** decreased. An increased trend was observed for ILT4 **(F)** and PD-L1 **(H)**. No changes were observed for OX40L expression **(G)**. CPE and scFOS/lcFOS (FF) did not alter expression levels of matured DCs. Data are presented as box plot summary. *P<0.05, **P<0.01, n=12.

To determine whether the NDO mixture scFOS/lcFOS or the peanut extract were able to alter the maturation status of DCs, DCs were either left untreated (imDC), matured (matDC), or matured in the presence of scFOS/lcFOS (FF), CPE or a combination of CPE and scFOS/lcFOS (CPE/FF) (**Figure 3**). DCs matured with the cytokine mix showed increased expression of maturation surface markers CD80 (**Figure 3A**), CD83 (**Figure 3B**), and CD86 (**Figure 3C**). Expression of DC-SIGN, a C-type lectin receptor surface molecule that recognizes mannose type carbohydrates and is mainly expressed on immature DCs (17, 18) was decreased (**Figure 3D**). ILT3 and ILT4 are membrane proteins with cytoplasmic immunoreceptor tyrosine-based inhibitory motifs (ITIMs) and are involved in the down-regulation of immune responses. ILT3 and ILT4 are mainly expressed by tolerogenic DCs (19, 20). ILT3 expression was decreased after maturation (**Figure 3E**) while ILT4 showed a tendency to increase (**Figure 3F**), which was significant in the presence of CPE during maturation. Expression of OX40L on DCs can activate cells expressing OX40, such as activated T cells (21). Enhanced expression of OX40L induces a Th2 cell–promoting effector DC (DC2) (22). In the experiments performed, no changes were observed in OX40L expression (**Figure 3G**). Lastly, PD-L1 expression of DCs was assessed, and showed a tendency to increase when DCs were matured, which was not affected by CPE and/or FF (**Figure 3H**). PD-L1 plays a role in proliferation of T cells and cytokine production; blocking PD-L1 resulted in enhanced T cell proliferation and an increase in IL-10 and IFN-γ (23). In conclusion, maturation of DCs resulted in increased expression of CD80, CD83, CD86, ILT4 and PD-L1, while decreasing DC-SIGN and ILT3. Exposure to FF or CPE did not further alter expression levels. This indicates that CPE and FF do not alter the maturation status of the DCs, or expression of tolerogenic or Th2-related expression markers.

### Production of IL-10 and IP-10 Is Enhanced in DCs Matured With CPE and FF

**Figure 4:**
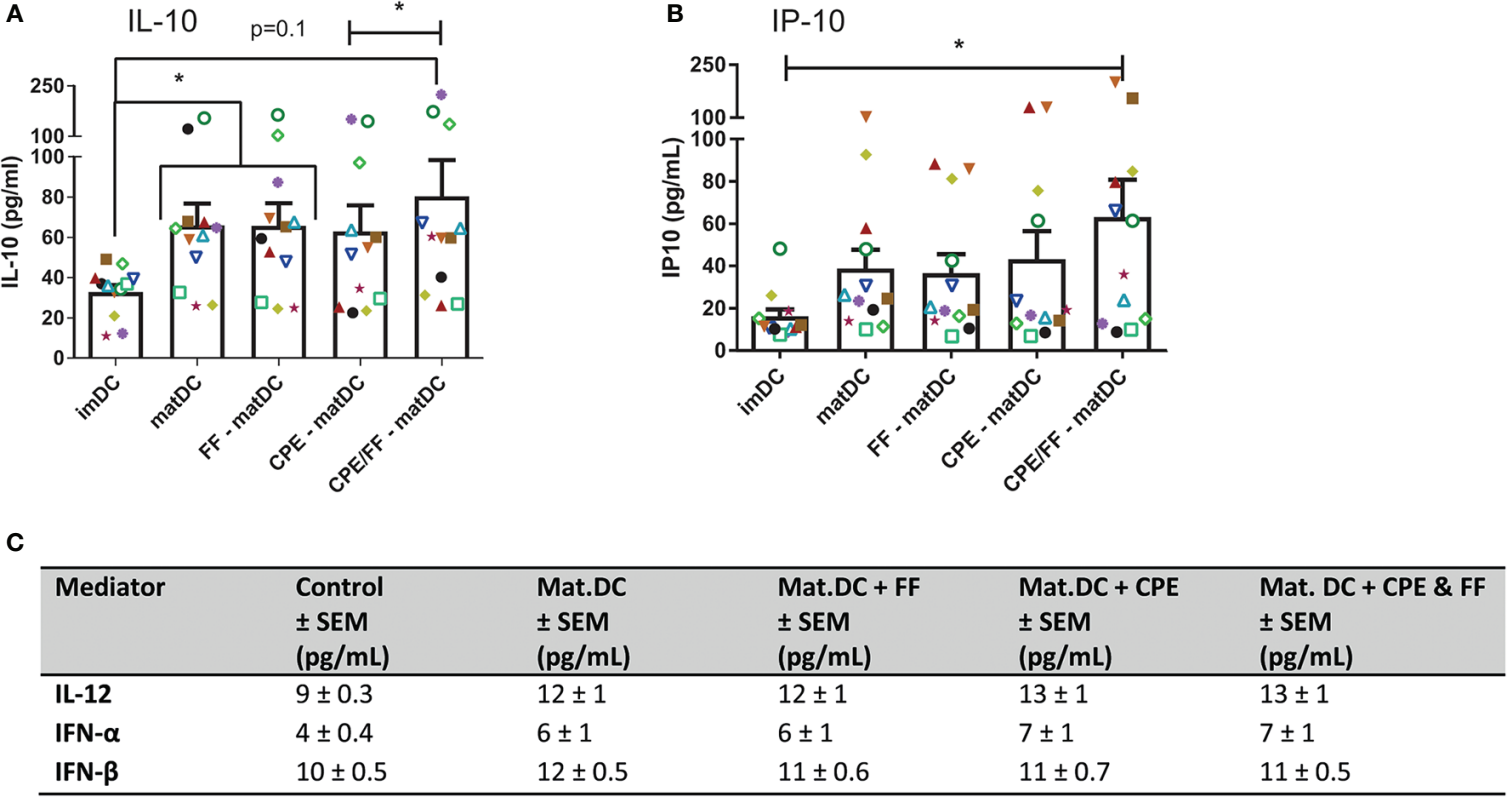
Cytokine production by DCs. After maturation, supernatant of DCs was harvested and analyzed by luminex. IL-10 produced by DCs increased when DCs were exposed to the maturation mix **(A)**. Although CPE and scFOS/lcFOS (FF) did not further enhance IL-10, the combination of CPE and FF increased IL-10 concentrations compared to CPE alone. IP-10 production by DCs increased after combined maturation with CPE and FF **(B)**. No effects of maturation, CPE and FF were observed for IL-12, IFN-α, and IFN-β **(C)**. Data are presented as mean ± SEM, *P<0.05, n=12. The different symbols and colors represent the measured values of independent peanut allergic donors.

After maturation, mediator production by DCs was analyzed (**Figure 4**). IL-10 concentrations increased significantly in matDCs in presence or absence of FF (**Figure 4A**), which might indicate a more tolerogenic DC phenotype. CPE exposure during DC maturation did not result in significantly increased IL-10 levels in DCs compared to matDCs. However, combining CPE with FF during maturation showed a significant increase in IL-10 compared to CPE alone, but was not different from IL-10 production by matDCs. In addition to an increase in IL-10, also production of Th1-associated chemokine IP-10 by DCs increased when DCs were matured with the combined presence of CPE and FF (**Figure 4B**), while maturation alone with or without only CPE or FF did not result in significant increases in IP-10. No differences were observed in IL-12, IFN-α, and IFN-β secretion in the supernatant (**Figure 4C**). This indicates that the combined exposure of DCs to both CPE and FF during maturation induces a phenotype with both regulatory and Th1-like features for instructing T cells.

### CPE/FF-matDCs Tend to Enhance Treg/Th1 and Treg/Th2 Ratios in an Autologous Peanut-Specific DC-T Cell Co-Culture

**Figure 5:**
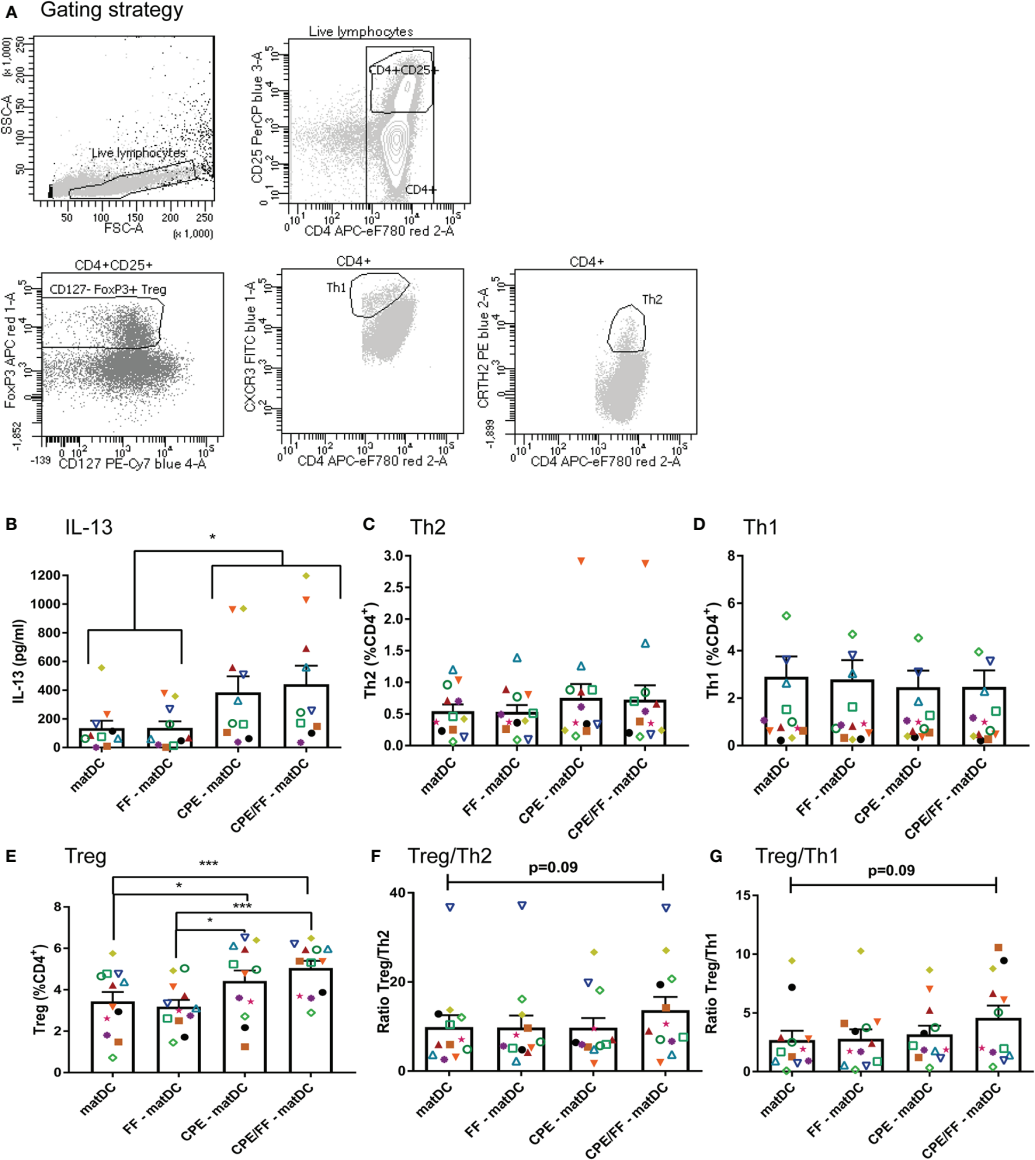
T cell polarization. Matured DCs were co-cultured for 6 to 7 days with autologous CD4^+^ T cells. Populations were identified as indicated **(A)**. Only T cells co-cultured with CPE-matured DCs secreted IL-13 **(B)**. Th2 polarization **(C)** and Th1 polarization **(D)** were not significantly affected. Treg polarization increased in the presence of CPE-matDCs +/− FF compared to matDCs and FF-matDCs **(E)**. CPE/FF-matDCs showed a trend in increasing the ratios between Treg/Th1 and Treg/Th2 **(F, G)**. Data are presented as mean ± SEM, *P<0.05, ***P<0.001. n=10 for IL-13 production (for 2 patients, IL-13 production was below the detection limit of the ELISA), n=12 for T cell polarization. The different symbols and colors represent the measured values of independent peanut allergic donors.

CPE and/or FF exposed matured DCs were co-cultured for 6 to 7 days with autologous CD4^+^ T cells. In this DC-T cell co-culture, IL-13 cytokine production, T cell polarization and proliferation were measured (**Figure 5**). T-cell subsets were gated as indicated (**Figure 5A**). Similar as described in **Figure 1**, only T cells that were co-cultured with CPE-matured DCs secreted significant amounts of IL-13 compared to the controls (**Figure 5B**). Th2 (CD4^+^CRTH2^+^) and Th1 (CD4^+^CXCR3^+^) polarization were not significantly affected upon CPE exposure of DCs in presence or absence of FF (**Figures 5C, D**). Treg polarization increased significantly in the presence of CPE-matDCs or CPE/FF-matDCs compared to matDCs without CPE (+/− FF) (**Figure 5E**). Overall, only DCs matured with both CPE and FF tended to increase Treg/Th1 and Treg/Th2 ratios (**Figures 5F, G**), indicating that combined exposure of DCs to CPE and FF during maturation may favor the T cell balance toward a more tolerogenic phenotype.

### Peanut-Induced T Cell Proliferation Is Enhanced When Co-Cultured With CPE +/− FF Exposed matDCs

**Figure 6:**
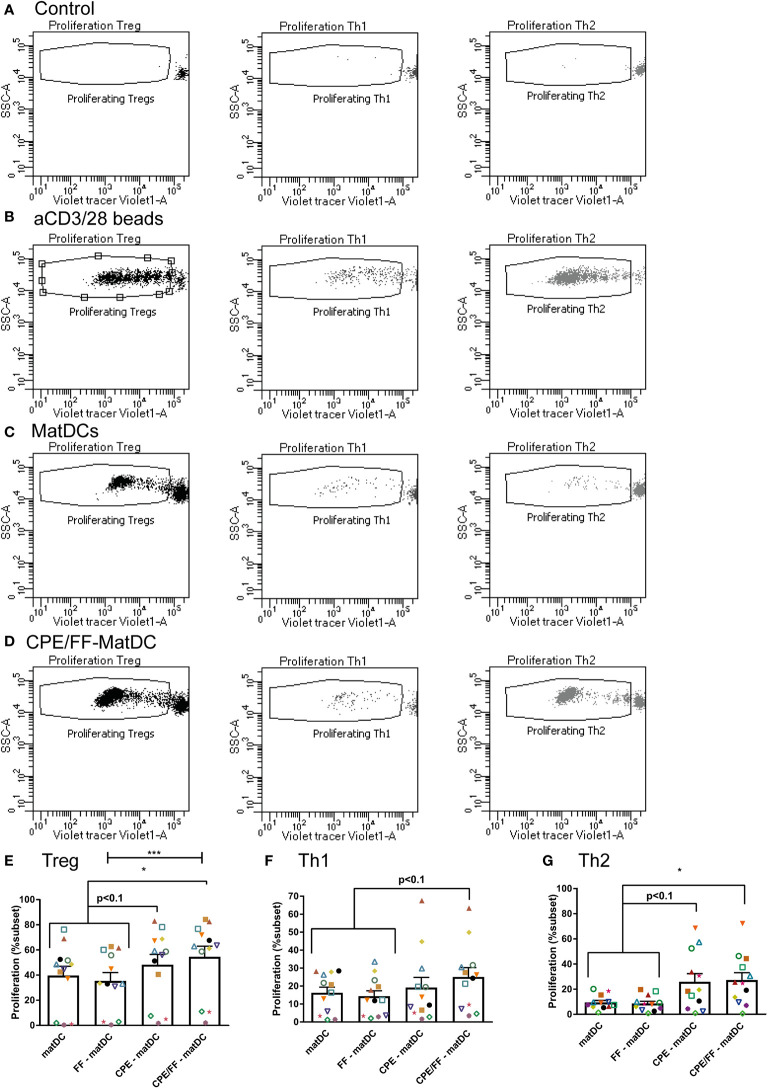
T cell proliferation Proliferation of the different subsets was identified as indicated **(A–D)**. Treg proliferation was enhanced after co-culture with CPE/FF-matDCs, and exposure to CPE during DC maturation showed a similar trend **(E)**. An increased trend in enhanced Th1 proliferation was also observed in T cells co-cultured with CPE/FF-matDCs **(F)**. Th2 proliferation was significantly enhanced by co-culture with CPE/FF-matDCs, while co-culture with CPE-matDCs showed the same trend **(G)**. Data are presented as mean ± SEM, *P<0.05, ***P<0.001, n=12. The different symbols and colors represent the measured values of independent peanut allergic donors.

In addition to T cell polarization, the corresponding T cell proliferation was assessed (**Figure 6**). Cell subsets were gated as in **Figure 5**, and the proliferation of these subsets was measured as indicated (**Figures 6A–D**). Controls included were T cells co-cultured with imDCs (**Figure 6A**), which did not show any proliferation as expected (since these DCs were not matured and do not present CPE to the T cells) and T cells stimulated with aCD3/28 beads (**Figure 6B**) to induce a full generic proliferative response. In addition, representative T cell proliferation of one patient was shown for Th cells co-cultured with matDCs (**Figure 6C**) or Th cells co-cultured with CPE/FF-matDCs (**Figure 6D**). Similar to Treg polarization, Treg proliferation was also significantly enhanced after co-culture of autologous Th cells of peanut allergic patients with CPE/FF-matDCs compared to matDCs (+/− FF), while for CPE-matDCs a similar trend was shown (**Figure 6E**). In addition, these CPE/FF-matDCs tended to induce Th1 proliferation compared to matDCs (**Figure 6F**). Th2 proliferation (**Figure 6G**) in the presence of CPE-matDCs tended to increase compared to matDCs (+/− FF), which became significant upon co-culture of Th cells with CPE/FF-matDCs. Furthermore, survival of T cells as assessed by the live lymphocyte gate was not affected by the exposure to CPE or FF (data not shown).These proliferation studies indicate that the T cells of peanut-allergic patients proliferated upon exposure to CPE presented by their DCs, indicating these Th cell responses to be allergen-specific. Exposure of DCs to FF during CPE-specific maturation was not able to reduce Th2 proliferation, however also Treg proliferation enhanced and Th1 proliferation showed the same tendency.

## Discussion

The direct influence of prebiotic mixture scFOS/lcFOS on maturation of immature moDCs and subsequent antigen presentation to autologous CD4^+^ T cells in a peanut-specific manner was investigated in this study. imDCs exposed to CPE were not able to induce a peanut-specific response by autologous T cells (hallmarked by IL-13), whereas DC maturation with a cytokine mix and combined exposure to CPE did induce an allergen-specific response. In this allergen specific model, the most important findings are that combined exposure to CPE and FF during DC maturation can induce a more tolerogenic/DC1 phenotype hallmarked by increased production of IL-10 and IP-10 in presence of FF. Although IL-10 production was already increased in matured DCs, FF significantly further increased the IL-10 production of CPE-matDCs. However, the question remains whether this increase in IL-10 by FF has further functional effects on the outcome of the T cell response. For future experiments, it would be interesting to neutralize IL-10 to determine if IL-10 released by FF exposed CPE-matDCs is involved in the indicated tendency toward enhancing the peanut specific Treg/Th2 and Treg/Th1 ratio as was shown in the autologous matDC/T-cell co-culture. Despite these effects the allergen-specific response in terms of CPE-matDCs induced IL-13 secretion by autologous T-cells and enhanced Th2 proliferation could not be decreased by the presence of FF during CPE-matDC generation. However, overall peanut specific Treg proliferation significantly increased, while Th1 polarization showed the same tendency when compared to matDCs when DCs were exposed to both CPE and FF during maturation, and a similar trend for Treg and Th2 proliferation was observed for CPE exposure alone. These findings indicate that during allergen-specific maturation of DCs FF might help to tip the balance toward a more Treg and Th1 cell prone immune response, even though Th2-specific proliferation and cytokines were not affected.

To the best of our knowledge this is the first autologous DC-T cell co-culture using cells of peanut-allergic patients that shows a peanut-specific response in terms of allergen-specific IL-13 secretion, Th2 proliferation, and Treg polarization and proliferation. Although other studies have been performed with similar co-culture models using peanut (24), these studies focused mainly on proliferation rather than polarization and cytokine production. Intracellular production of IL-4 could be measured, but no differences were observed between healthy donors and allergic patients. Other co-culture studies focusing on food allergens in DC-T cell co-cultures were also unable to measure cytokines, such as IL-13 (25). This might be related to the maturation of the dendritic cells. In the latter studies, DCs were pulsed only with food allergen, without the addition of maturation cytokines. When the concentration of allergen-extract is high enough, an induction in maturation status of the DCs was observed, however this could not be translated into an allergen-specific response by T cells. In contrast, a study using inhalant allergens was also able to induce an allergen-specific response in T cells of poly-sensitized individuals. Here, inhalant allergen-pulsing was combined with maturation of moDCs using TNF-α and IL-1β (26). In contrast to a recent study where direct effects of a different prebiotic mixture scGOS/lcFOS were observed (11), we were not able to find immunomodulatory effects of scFOS/lcFOS when directly added during maturation of the DCs without further maturation with cytokines. This can be related to the concentration of scFOS/lcFOS used in this study which was lower and approaches physiological circulating levels (8–10). A limitation of this study was that due to the limited amount of patient material, we were not able to compare scFOS/lcFOS with the previously studied scGOS/lcFOS and could unfortunately not test the patient-specific response to the specific peanut allergens (Ara h 1, 2, 3, or 6) in the DC-T cell model described in the study. Although we did observe CPE-specific IL-13 secretion and proliferation of Th2 cells (**Figure 6**), future studies would need to further address the allergen-specificity of this model either with non-allergic patients or an endogenous control challenge.

It appears that the combination of scFOS/lcFOS and CPE during DC maturation plays an important role in the outcome of parameters involved in T cell polarization and proliferation. Where scFOS/lcFOS alone did not modulate the DC maturation status, only in CPE exposed matDCs FF facilitated the enhance in IL-10 secretion by the matDCs instructing T cell polarization toward a more Treg prone phenotype as indicated by a tendency toward increased Treg/Th2 and Treg/Th1 ratios. Although peanut induced cytokine production of IL-10 and IFN-γ by T cells could not be detected, while IL-13 secretion remained unaltered high, this might indicate a supportive role for scFOS/lcFOS in phenotypic changes of allergen exposed DC maturation under inflammatory conditions. Indeed in a murine cow’s milk allergy model, where oral immunotherapy (OIT) was combined with a diet of scFOS/lcFOS, an increase in the Treg population in the mesenteric lymph nodes (MLN) was observed and the efficacy of OIT was enhanced by scFOS/lcFOS (27). In addition, a different oligosaccharide mixture scGOS/lcFOS also was able to induce Tregs in an allogeneic DC-T cell culture with healthy donors albeit at a much higher dosage (11).

Unfortunately, the direct mechanism of action for scFOS/lcFOS has not been elucidated. Type-I IFN production by matDCs was not increased after exposure to scFOS/lcFOS. This indicates that scFOS/lcFOS does not act *via* the Dectin-1 receptor, since activation of this receptor is known to enhance type-I IFN release by DCs. Also previous research in our group indicated that neutralizing Dectin-1 did not affect the immunomodulatory effects of FF (28). In another study, a possible mechanism *via* TLR4 was proposed, where a high dosage of scGOS/lcFOS induced IL-10 release by moDCs which was abrogated in the presence of a TLR4 antagonist (11). Unfortunately, due to restrictions in patient material, we were not able to study this in the current peanut-specific model. A recent study postulated that the observed effects of these oligosaccharides *via* TLR4 most likely can be ascribed to contamination of the samples with LPS (29). Although previous effects of scGOS/lcFOS resulted from a solution with endotoxin levels lower than 3 ng/ml (11), the study of Perdijk et al. indicated that levels of >0.5 EU/ml (1EU ~0.1 ng endotoxin) already can influence DCs and can result in tolerogenic DCs. The oligosaccharide mixture that was used in this study was also analysed for endotoxin content by means of a LAL-assay. The measured endotoxin content of these mixtures was <0.07 EU/ml in the concentration used and therefore we believe that the observations in this study cannot be ascribed to endotoxin contamination.

The current study shows the development of a novel model to study allergen specific autologous DC/T-cell interactions. This model can be further used and expanded to study mechanisms of immunomodulatory components, not only in allergies but also for example in vaccination responses. In this respect, it should be realized that for this study a DC2 driving cytokine mix was used to mature the moDC of the peanut allergic patients, since this is most appropriate in an allergic setting. Due to restriction in study material we could not compare the outcome with the general used DC1 maturation component LPS, yielding another outcome of the T-cell response (30). Furthermore, when studying the role for dietary components or bacterial fermentation products in these type of models, it may be considered to also include the cross-talk between intestinal epithelial cells and the underlying dendritic cells. This is of relevance since the latter components may instruct the intestinal dendritic cell directly or indirectly *via* alteration of the function and mediator release of the epithelium. The latter may also contribute to the modulation of the DC phenotype and functional outcome of the T-cell response (31).

In conclusion, in a peanut-specific autologous DC-T cell co-culture assay, the addition of scFOS/lcFOS during maturation of CPE-exposed DCs enhanced regulatory and Th1-related mediator release and subsequently tended to increase the Treg/Th2 and Treg/Th1 ratios. scFOS/lcFOS might alter the DC phenotype during maturation in presence of the allergen and in this way modify the balance of the successive T cell response toward a more Treg and Th1 prone direction, albeit this effect was not strong enough to suppress peanut induced Th2 proliferation and cytokine production. This study indicates that beyond its known function as prebiotic, scFOS/lcFOS also is capable of directly modulating DC function making it an interesting candidate as adjunct treatment for allergen specific immunotherapeutic approaches in food allergy.

## Data Availability Statement

The raw data supporting the conclusions of this article will be made available by the authors, without undue reservation.

## Ethics Statement

The studies involving human participants were reviewed and approved by Ethics Committee of the University Medical Center Utrecht (NL51606.041.15). The patients/participants provided their written informed consent to participate in this study.

## Author Contributions

SH, LW, and HO designed the experiments. AK assisted in recruitment of patients. SH performed the experimental procedures. SH performed data collection and analyses and drafted the manuscript. JG, AK, LW, and HO contributed to data interpretation and critically revised the manuscript. All authors contributed to the article and approved the submitted version.

## Funding

This project was funded by a Dutch Government NWO/STW Funding, project number 12652 NUTRALL (Nutrition-based approach to support antigen-specific immunotherapy for food allergies).

## Conflict of Interest

JG is employed at the Utrecht University and at Nutricia Research B.V. LW works at the Pharmacology division of the Utrecht University within a strategic alliance with Nutricia Research B.V.

The remaining authors declare that the research was conducted in the absence of any commercial or financial relationships that could be construed as a potential conflict of interest.

## Supplementary Material

Supplementary Figure 1 Effect of scFOS/lcFOS during maturation or differentiation and maturation (d) scFOS/lcFOS (FF) was added to DCs during the two days of maturation in the presence of CPE or during differentiation (d) and maturation in combination with CPE. No differences were found in time-point of addition of scFOS/lcFOS on T cell polarization **(A)**, or T cell proliferation **(B)**, n=5. The different symbols and colors represent the measured values of independent peanut allergic donors.
