# Supplementary figures and images for: Fructo-Oligosaccharides Modify Human DC Maturation and Peanut-Induced Autologous T-Cell Response of Allergic Patients In Vitro
Source: Front Immunol. 2021 Feb 15;11:600125. doi: 10.3389/fimmu.2020.600125 (PMC7917053; doi:10.3389/fimmu.2020.600125)

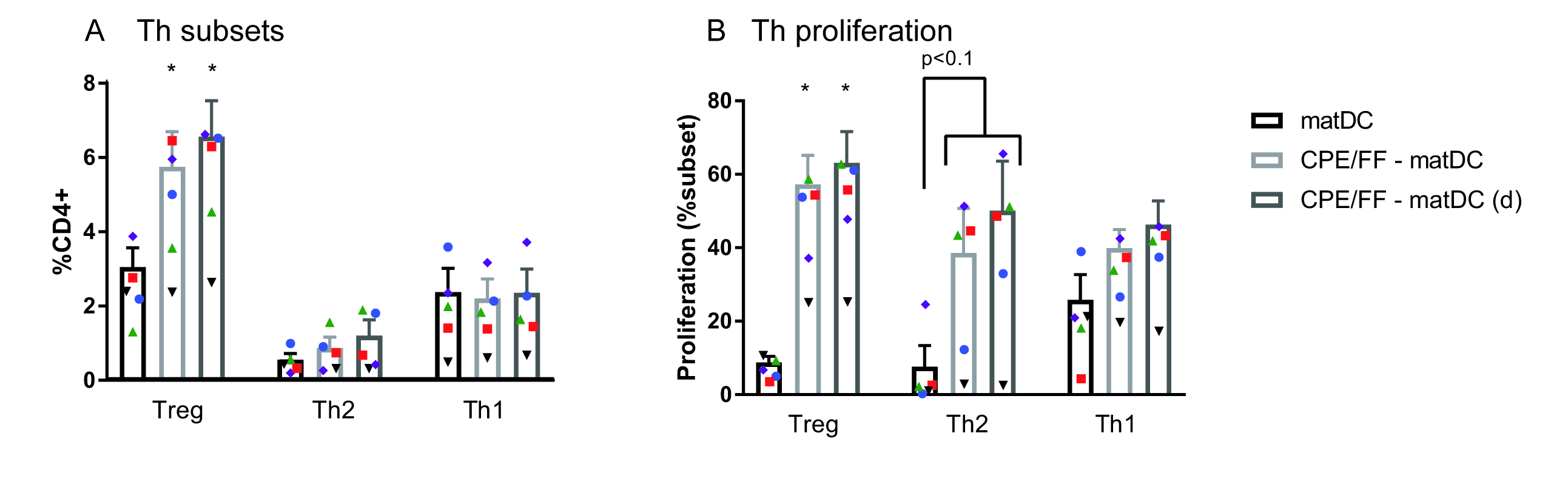

Supplement: Supplementary Figure 1 — Effect of scFOS/lcFOS during maturation or differentiation and maturation (d) scFOS/lcFOS (FF) was added to DCs during the two days of maturation in the presence of CPE or during differentiation (d) and maturation in combination with CPE. No differences were found in time-point of addition of scFOS/lcFOS on T cell polarization (A), or T cell proliferation (B), n=5. The different symbols and colors represent the measured values of independent peanut allergic donors. [file Image_1.tif]
